# Supplementary material for: DifferentialRegulation: a Bayesian hierarchical approach to identify differentially regulated genes
Source: bioRxiv. 2023 Aug 17:2023.08.17.553679. Preprint. [Version 1] doi: 10.1101/2023.08.17.553679 (PMC10462127; doi:10.1101/2023.08.17.553679)
Supplement: Supplementary file 1 [file media-1.pdf]

Supplementary Material
DifferentialRegulation: a Bayesian hierarchical approach to identify differentially regulated genes

Simone Tiberi[1,2*], Joël Meili[2†], Peiying Cai[2†], Charlotte Soneson[3†], Dongze He[8,9], Hirak Sarkar[5], Alejandra Avalos-Pacheco[6,7], Rob Patro[4,9], and Mark D Robinson[2]

[1]*Department of Statistical Sciences, University of Bologna, Bologna, Italy.*
[2]*Department of Molecular Life Sciences and SIB Swiss Institute of Bioinformatics, University of Zurich, Zurich, Switzerland.*
[3]*Computational Biology Platform, Friedrich Miescher Institute for Biomedical Research and SIB Swiss Institute of Bioinformatics, Basel, Switzerland*
[4]*Department of Computer Science, University of Maryland, MD, USA*
[5]*Department of Computer Science, Princeton University, NJ, USA*
[6]*Research Unit of Applied Statistics, TU Wien, Vienna, Austria*
[7]*Harvard-MIT Center for Regulatory Science, Harvard Medical School, Boston, MA, USA*
[8]*Department of Cell Biology and Molecular Genetics, University of Maryland, MD, USA*
[9]*Center for Bioinformatics and Computational Biology, University of Maryland, MD, USA*
[†]these authors contributed equally. [*]e-mail: Simone.Tiberi@unibo.it

1 Supplementary Details

1.1 Ambiguous reads in single-cell RNA-seq data

When using single-cell RNA-seq data, we only employ a latent variable approach for multi-gene mapping reads, while a reads are not allocated, and are treated separately from s and u. This is because, in order to allocate a reads, we need an estimate of the probability that an ambiguous read is spliced (or unspliced), which cannot be accurately computed, because it depends on various unknown factors.

In fact, existing single-cell analysis tools either assign all ambiguous reads as spliced, for example, *velocyto* (La Manno et al., 2018), or acknowledge the difficulty and quantify the result of the ambiguous reads as a stand-alone count matrix (i.e., a), and leave the task of solving the ambiguity to the users, such as *alevin* (Srivastava et al., 2019) and *alevin-fry* (He et al., 2022). The difficulty of ambiguous read allocation comes from both biological and computational aspects. First, s and u mRNA share large genomic regions (e.g., exons), while scRNA-seq reads are characterized by short fragments, which are often compatible with both splice variants of genes. Second, most ambiguous reads can be allocated to spliced and unspliced genes equally well, with no decisive evidence for disambiguating those reads. Third, while read coverage is approximately uniform in bulk RNA-seq reads (i.e., the location of reads in a transcript can be assumed to be roughly random, hence uniform, across the transcript), this is not the case for single-cell protocols. At present, we still lack accurate methods that model the underlying mechanism of ambiguous reads, and address the respective uncertainty in a probabilistic way.

1.2 MCMC scheme

We alternately sample parameters and latent states from their conditional distributions, following a Metropolis-within-Gibbs sampling scheme. In particular, we sample: hyper-parameters $\delta|\pi$ from a Metropolis sampler,

with an adaptive random walk proposal; parameters $\rho|Y$ from Dirichlet Gibbs samplers; parameters $\pi|X, \delta$ from Dirichlet Gibbs samplers; latent states $X|\pi$ from multinomial Gibbs samplers. Below, we describe the prior specifications, and the details of each sampling scheme.

1.2.1 $\delta|\pi$ Metropolis sampling

We use an empirical Bayes approach to formulate an informative prior for the hyper-parameters. In particular, we randomly select 1,000 genes/transcripts, and, for each one of them, we use *DRIMSeq* (Nowicka and Robinson, 2016) to obtain an initial estimate of the following parameters: $log(\delta_+), log(\delta_S)$, and (in the single-cell model) $log(\delta_U)$. For each parameter, we compute the mean and standard-deviation of its 1,000 estimated values (1 per selected gene): μ_+ and σ_+ for $log(\delta_+)$, μ_S and σ_S for $log(\delta_S)$, and (in the single-cell model) μ_U and σ_U for $log(\delta_U)$.

These estimates are used to formulate the following informative prior distributions for the hyper-parameters: $log(\delta_+) \sim \mathcal{N}\left(\mu_+, \sigma_+^2\right)$, $log(\delta_S) \sim \mathcal{N}\left(\mu_S, \sigma_S^2\right)$, and (in the single-cell model) $log(\delta_U) \sim \mathcal{N}\left(\mu_U, \sigma_U^2\right)$.

Hyper-parameters are sampled, in the logarithmic scale, from a Metropolis sampler; in particular, we sample parameters $(log(\delta_S), log(\delta_U), log(\delta_A))$, (or simply $(log(\delta_S), log(\delta_U))$ in the bulk model), and, to obtain their prior distributions, apply the change of variable via the Jacobian transformation (Murphy, 2012).

Values are proposed based on an adaptive random walk (Haario et al., 2001). In the initial 200 iterations, we propose values from a normal distribution independently for each hyper-parameter. After 200 iterations, a covariance matrix of the posterior chains is computed, and hyper-parameters are proposed from a multivariate normal distribution, with this covariance matrix. The covariance matrix is then re-updated at 300, 400, 500 iterations, and at the burn-in (which is set to be at least 500 iterations).

1.2.2 $\rho|Y$ Gibbs sampling

We assume a weakly informative conjugate Dirichlet prior distribution for ρ,

$$\rho_i \sim Dir\left(1, \ldots, 1\right), \text{ for } i = 1, \ldots, N, \tag{1}$$

which results in the following posterior distributions:

$$\rho_i | Y_i \sim Dir\left(Y_i^{(1)} + 1, \ldots, Y_i^{(T)} + 1\right). \tag{2}$$

In the single-cell model, G (the number of genes) replaces T (the number of transcripts).

1.2.3 $\pi|X, \delta$ Gibbs sampling

As described in the main paper, parameters π have an informative conjugate Beta (bulk model) or Dirichlet (single-cell model) hierarchical prior distribution. The former case results in the following Beta posterior distribution:

$$\pi_{Si}^{(t)} | X_i, \delta \sim Beta\left(X_{Si}^{(t)} + \delta_S^{(t)}, X_{Ui}^{(t)} + \delta_U^{(t)}\right), \text{ for } t = 1, \ldots T; \tag{3}$$

the latter case, instead, leads to the following Dirichlet posterior distribution:

$$\pi_i^{(g)} | X_i, \delta \sim Dir\left(X_{Si}^{(g)} + \delta_S^{(g)}, X_{Ui}^{(g)} + \delta_U^{(g)}, X_{Ai}^{(g)} + \delta_A^{(g)}\right), \text{ for } g = 1, \ldots G. \tag{4}$$

1.2.4 $X|pi$ Gibbs sampling

Assume that a total of J equivalence classes (EC) are available. Each EC is defined by two elements, which for the j-th EC are W_j and Z_j: the former contains the list of gene(s)/transcript(s) compatible with the j-th equivalence class, while the latter indicates the splice status of the corresponding gene(s)/transcript(s) (i.e., s or u for bulk data, and s, u or a for single-cell data). Each EC is also associated to a count, c_j for the j-th class, indicating the number of (bulk or sc) RNA-seq reads compatible with it.

For each EC, we aim to allocate its counts to the gene(s)/transcript(s), and respective splice status, compatible with it.

For simplicity, below we consider the bulk model; the single-cell model is a natural extension of it. We define $X_{iSj}^{(t)}$ and $X_{iUj}^{(t)}$ as the contribution (i.e., counts) of the j-th EC to the spliced and unspliced versions of transcript t in sample i.

Assume that the j-th EC is compatible with n_j transcripts; the counts of each EC are allocated according to the following multinomial distribution:

$$\left(X_{z_1 i}^{(w_1)}, \ldots, X_{z_{n_j} i}^{(w_{n_j})}, \right) \sim \mathcal{MN}\left(\pi_{ij}, c_j\right), \tag{5}$$

where $\pi_{ij} \propto \left(\rho_i^{(w_1)} \times \tilde{\pi}_{z_1 i}^{(w_1)}, \ldots, \rho_i^{(w_{n_j})} \times \tilde{\pi}_{z_{n_j} i}^{(w_{n_j})} \right)$, $W_j = \left(w_1, \ldots, w_{n_j}\right)$, and $Z_j = \left(z_1, \ldots, z_{n_j}\right)$, with $w_1, \ldots, w_{n_j} \in \{1, \ldots, T\}$, and $z_1, \ldots, z_{n_j} \in \{S, U\}$.

Once the counts of all J ECs have been allocated, we can recover the overall s and u abundance of each gene, by adding elements across ECs: $X_{iS}^{(t)} = \sum_{j=1}^{J} X_{iSj}^{(t)}$ and $X_{iU}^{(t)} = \sum_{j=1}^{J} X_{iUj}^{(t)}$; then, the overall transcript-level counts can be retrieved as $Y_i^{(t)} = X_{iS}^{(t)} + X_{iU}^{(t)}$.

In order to accelerate the runtime of our method, latent states are only sampled every 10 iterations (i.e., undersampling scheme).

1.3 Wald-type test for the single-cell model

Given two groups of samples, A and B, we aim to test if parameter π_S, π_U, and π_A change between groups. We consider the difference of these parameters across groups as: $\omega = (\omega_S, \omega_U)$, with $\omega_S = {}_A\pi_S - {}_B\pi_S$, and $\omega_U = {}_A\pi_S - {}_B\pi_S$. Under the null hypothesis, π_S and π_U (and consequently π_A) do not change across groups; therefore we test the following system of hypotheses: $H_0 : \omega_S = \omega_U = 0$; H_1 : otherwise.

We approximate the posterior distribution of ω with a bivariate normal (Gelman et al., 1995): $\omega \dot{\sim} \mathcal{N}(\hat{\omega}, \hat{\Sigma}_\omega)$, with parameters $\hat{\omega}$ and $\hat{\Sigma}_\omega$ inferred from the posterior chains. Given this normal approximation, we use a bivariate Wald test (Li et al., 1991): $\hat{\omega} \hat{\Sigma}_\omega^{-1} \hat{\omega}^T \dot{\sim} \chi_2^2$.

1.4 Simulation details

In the simulation study, we only simulate DR effects when the difference in the relative abundance of unspliced reads, $\bar{\pi}_U$, is at least 0.2 between groups (i.e., ${}_B\bar{\pi}_U - {}_A\bar{\pi}_U \geq 0.2$). This presents us from simulating negligible differential effects, which would be uninteresting.

When simulating DGE genes/transcripts, we randomly sample the fold change between conditions as $FC = 2+Z$, with $Z \sim exp(\mu = 1)$, where $exp(\mu)$ is the exponential random variable with mean μ. This generates random fold changes with mean 3, and variance 1.

DAS genes are obtained by inverting the abundances (i.e., counts) of the two most abundant transcripts in the gene. This is coherent with previous simulation studies involving DAS (Tiberi and Robinson, 2020; Soneson et al., 2016).

2 Supplementary Tables

cell type	number of cells
oRG	6776
CPNs	6361
Immature CPNs	5983
Immature PNs	2954
Cycling	2389
RG	1093

Supplementary Table 1: Number of cells available, across all six brain organoids, for each cell type, in the real data analysis study.

3 Supplementary Figures

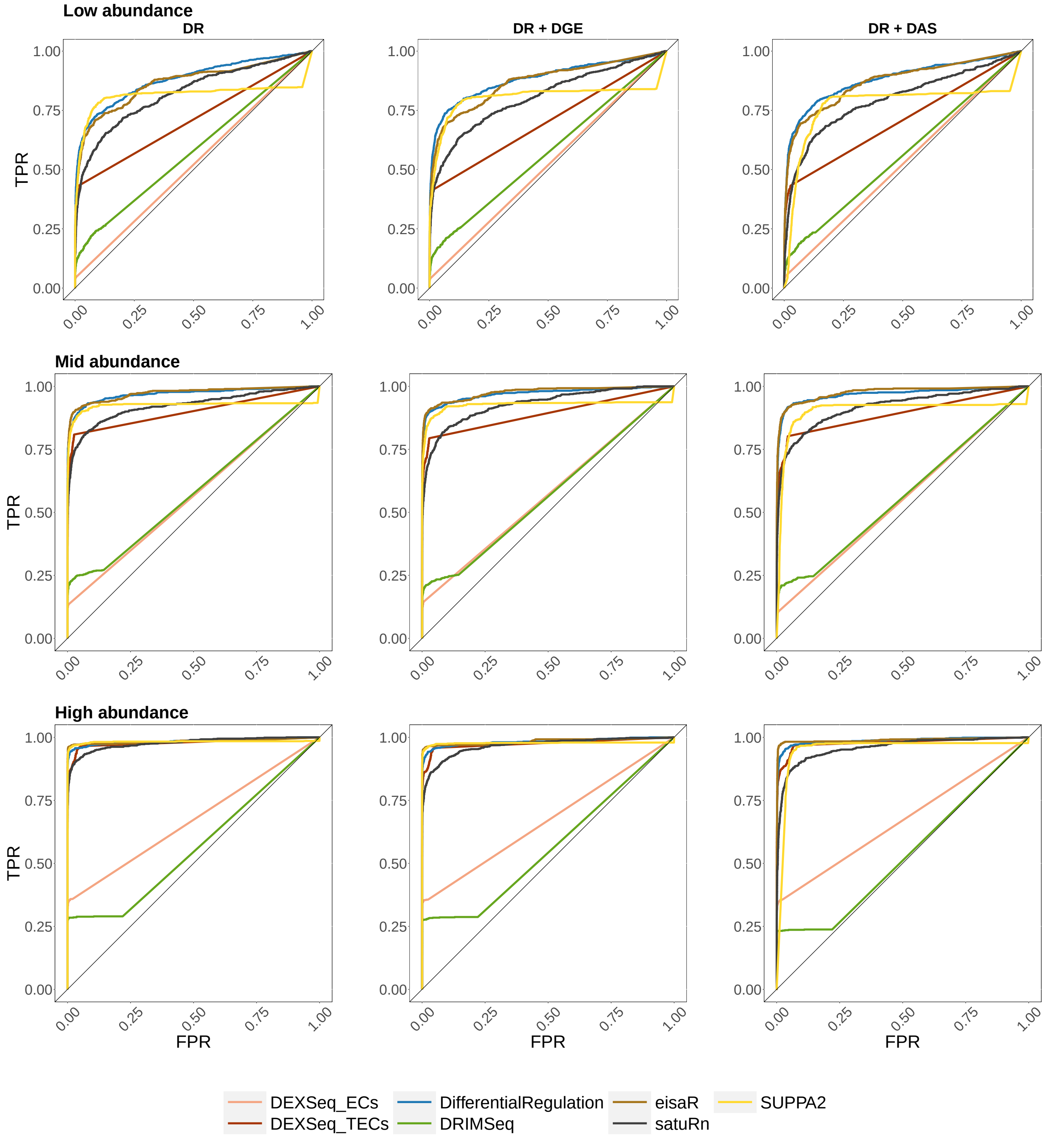

Supplementary Figure 1: ROC curves for the bulk simulations, stratified by overall transcript abundance: false positive rate (FPR) *vs.* true positive rate (TPR). Top row: low abundance (bottom 33% of abundant transcripts); middle row: medium abundance (middle 33% of abundance); bottom row: high abundance (top 33% of abundant transcripts). Left column: DR only simulation; middle column: DR and DGE simulation; right column: DR and DAS simulation.

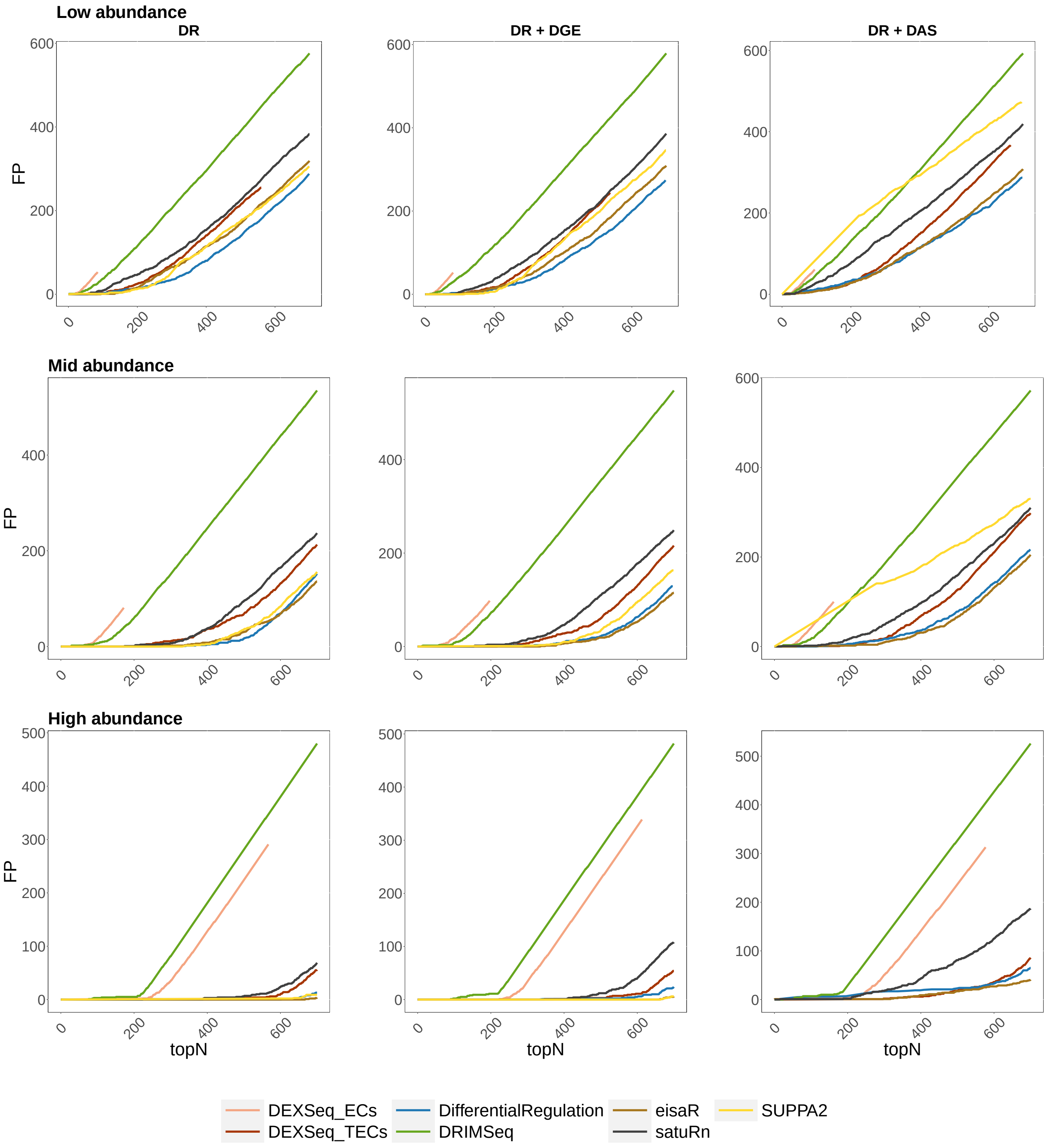

Supplementary Figure 2: False positive (FP) results among top detections (topN), stratified by overall transcript abundance: false positive rate (FPR) *vs.* true positive rate (TPR). Top row: low abundance (bottom 33% of abundant transcripts); middle row: medium abundance (middle 33% of abundance); bottom row: high abundance (top 33% of abundant transcripts). Left column: DR only simulation; middle column: DR and DGE simulation; right column: DR and DAS simulation.

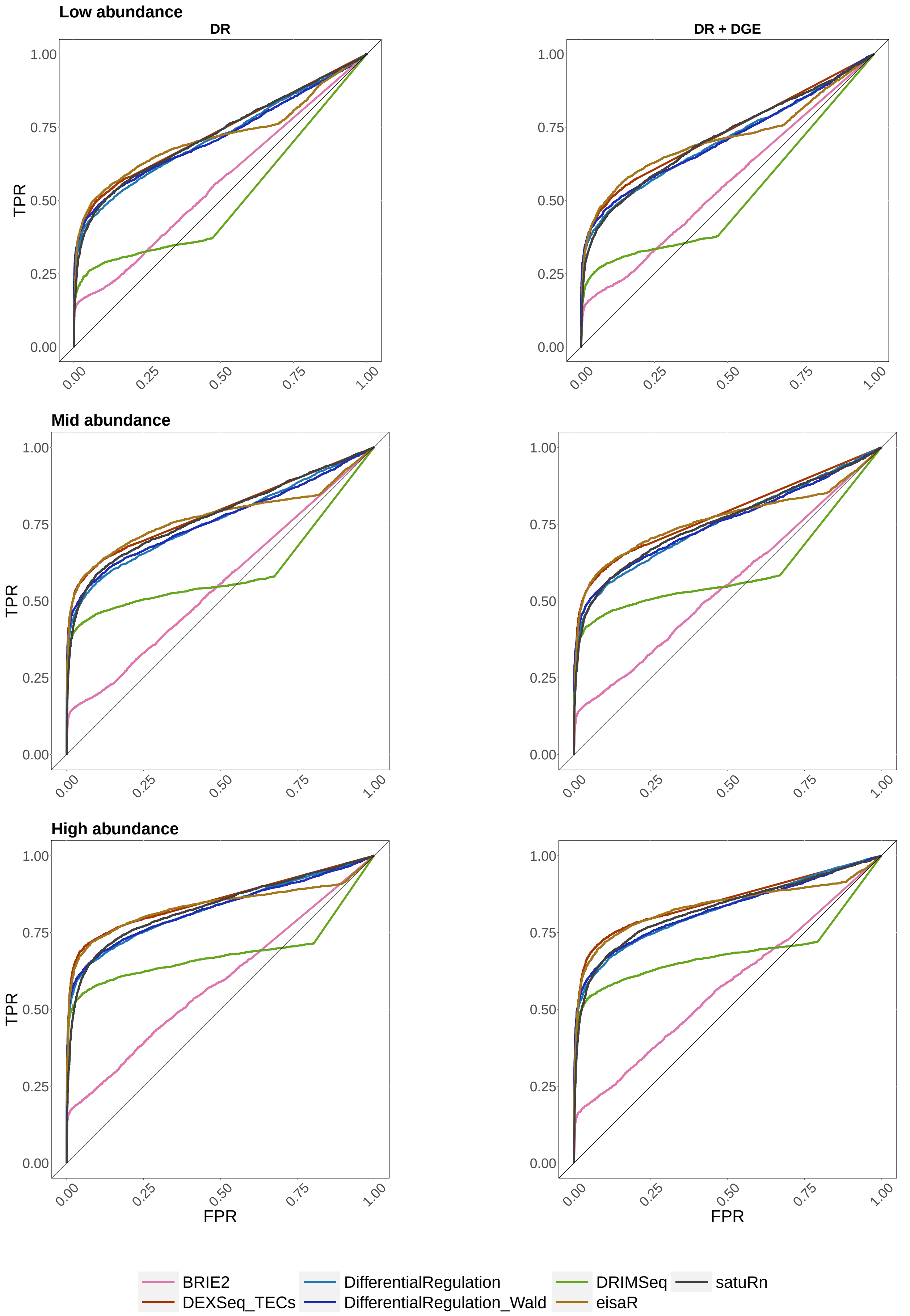

Supplementary Figure 3: ROC curves for the scRNA-seq simulations, stratified by overall transcript abundance: false positive rate (FPR) *vs.* true positive rate (TPR). Top row: low abundance (bottom 33% of abundant transcripts); middle row: medium abundance (middle 33% of abundance); bottom row: high abundance (top 33% of abundant transcripts). Left column: DR only simulation; right column: DR and DGE simulation.

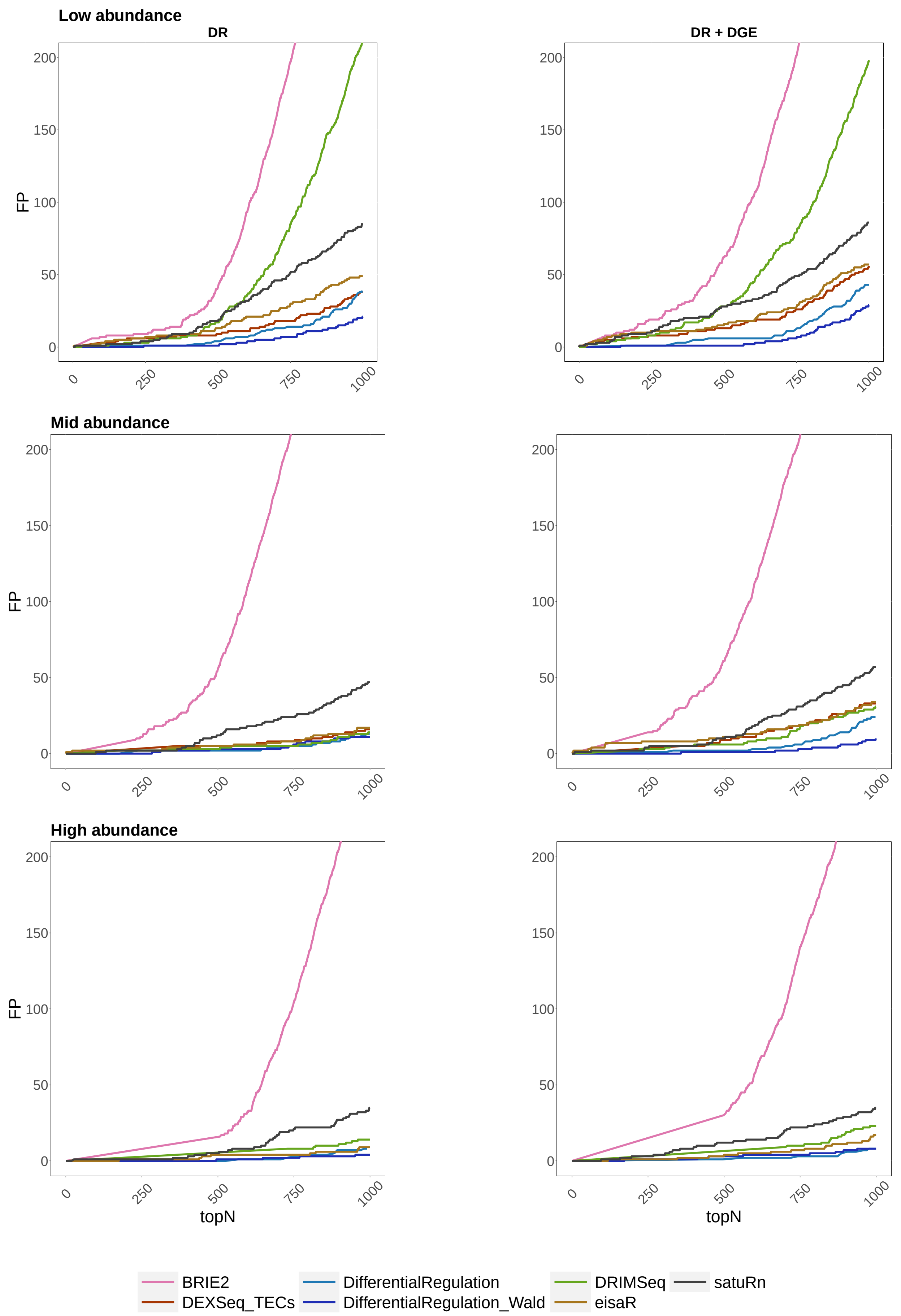

Supplementary Figure 4: False positive (FP) results among top detections (topN), for the scRNA-seq simulations, stratified by overall transcript abundance: false positive rate (FPR) *vs.* true positive rate (TPR). Top row: low abundance (bottom 33% of abundant transcripts); middle row: medium abundance (middle 33% of abundance); bottom row: high abundance (top 33% of abundant transcripts). Left column: DR only simulation; right column: DR and DGE simulation.

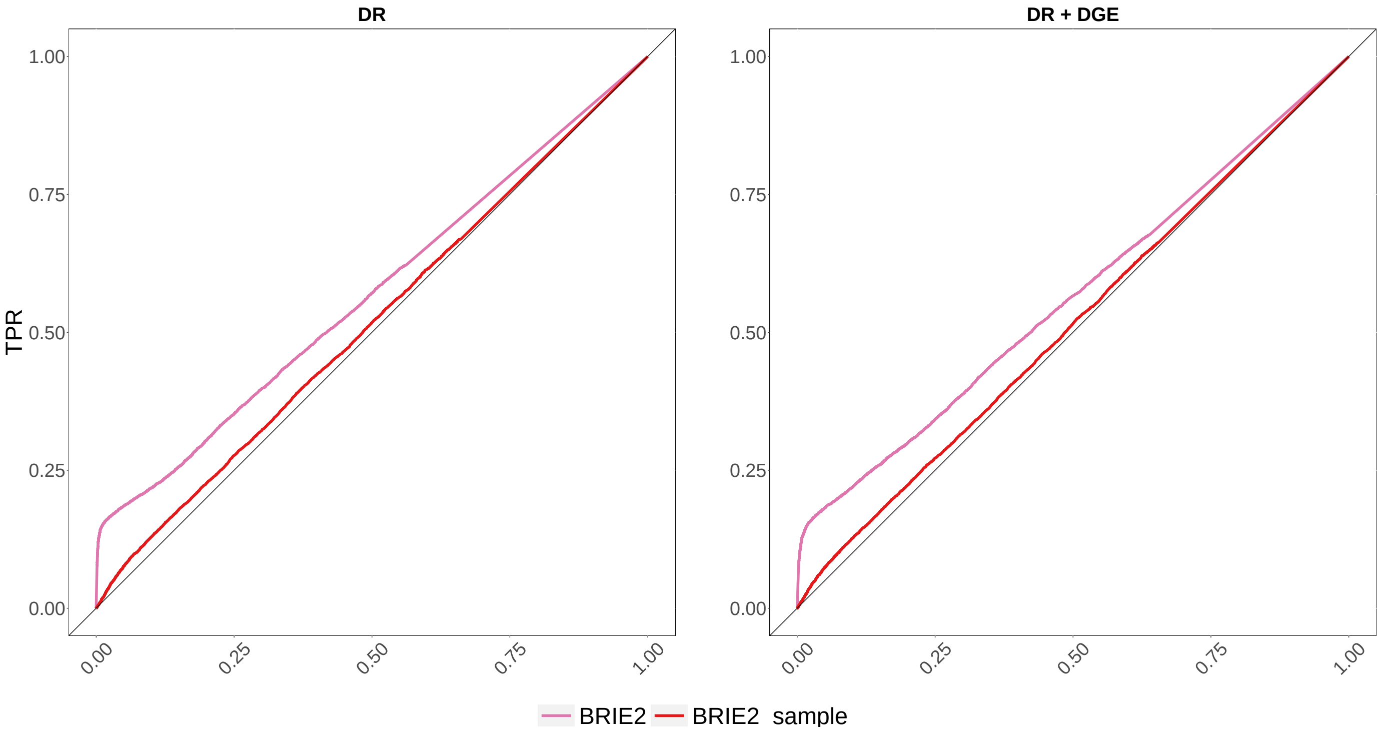

Supplementary Figure 5: ROC curves for the scRNA-seq simulations: false positive rate (FPR) *vs.* true positive rate (TPR). Here, we focus on *BRIE2*, with (*BRIE2_ sample*) and without (*BRIE2*) sample as a covaraite in the regression model. Left column: DR only simulation; right column: DR and DGE simulation.

References

A. Gelman, J. B. Carlin, H. S. Stern, and D. B. Rubin. *Bayesian data analysis.* Chapman and Hall/CRC, 1995.

H. Haario, E. Saksman, and J. Tamminen. An adaptive Metropolis algorithm. *Bernoulli*, pages 223–242, 2001.

D. He, M. Zakeri, H. Sarkar, C. Soneson, A. Srivastava, and R. Patro. Alevin-fry unlocks rapid, accurate and memory-frugal quantification of single-cell RNA-seq data. *Nature Methods*, 19(3):316–322, 2022.

G. La Manno, R. Soldatov, A. Zeisel, E. Braun, H. Hochgerner, V. Petukhov, K. Lidschreiber, M. E. Kastriti, P. Lönnerberg, A. Furlan, et al. RNA velocity of single cells. *Nature*, 560(7719):494–498, 2018.

K.-H. Li, T. E. Raghunathan, and D. B. Rubin. Large-sample significance levels from multiply imputed data using moment-based statistics and an F reference distribution. *Journal of the American Statistical Association*, 86(416):1065–1073, 1991.

K. P. Murphy. *Machine learning: a probabilistic perspective.* MIT press, 2012.

M. Nowicka and M. D. Robinson. DRIMSeq: a Dirichlet-multinomial framework for multivariate count outcomes in genomics. *F1000Research*, 5, 2016.

C. Soneson, K. L. Matthes, M. Nowicka, C. W. Law, and M. D. Robinson. Isoform prefiltering improves performance of count-based methods for analysis of differential transcript usage. *Genome biology*, 17:12, 2016.

A. Srivastava, L. Malik, T. Smith, I. Sudbery, and R. Patro. Alevin efficiently estimates accurate gene abundances from dscRNA-seq data. *Genome biology*, 20: 1–16, 2019.

S. Tiberi and M. D. Robinson. BANDITS: Bayesian differential splicing accounting for sample-to-sample variability and mapping uncertainty. *Genome Biology*, 21:1–13, 2020.
